# Effect of Synthetic Wax on the Rheological Properties of Polymer-Modified Bitumen

**DOI:** 10.3390/ma18133067

**Published:** 2025-06-27

**Authors:** Marek Iwański, Małgorzata Cholewińska, Grzegorz Mazurek

**Affiliations:** Department of Transportation Engineering, Faculty of Civil Engineering and Architecture, Kielce University of Technology, Al. Tysiąclecia Państwa Polskiego 7, 25-314 Kielce, Poland; m.cholewinska@tu.kielce.pl (M.C.); gmazurek@tu.kielce.pl (G.M.)

**Keywords:** polymer-modified bitumen, synthetic wax, rheological properties, bitumen aging, morphology

## Abstract

The goal of this study is to evaluate how the inclusion of synthetic wax, added in 0.5% increments from 1.5% to 3.5%, affects the characteristics of PMB 45/80-65 (polymer-modified bitumen) during both short-term (RTFOT) and long-term (PAV) aging processes. Tests were carried out to assess the fundamental properties of the binder, leading to the determination of the penetration index (PI) and the plasticity range (PR). The binder’s properties were examined at below-freezing operating temperatures, with creep stiffness measured using a bent beam rheometer (BBR) at −10 °C, −16° C, −22 °C, and −28 °C. The rheological properties of the asphaltenes were evaluated based on both linear and nonlinear viscoelasticity. The experimental study explored temperature effects on the rheological properties of composite materials using a DSR dynamic shear rheometer at 40 °C, 60 °C, and 80 °C over a frequency range of 0.005 to 10 Hz. The main parameters of interest were composite viscosity (η*) and zero shear viscosity (η_0_). Viscoelastic parameters, including the dynamic modulus (G*) and phase shift angle (δ), were determined, and Black’s curves were used to illustrate the relationship between these parameters, where G*/sinδ was determined. The MSCR test was employed to investigate the impact of bitumen on the asphalt mixture’s resistance to permanent deformation and to assess the degree and efficacy of asphalt modification. The test measured two parameters, irreversible creep compliance (J_nr_) and recovery (R), under stress levels of 0.1 kPa (LVE) and 3.2 kPa (N-LVE). The Christensen–Anderson–Marasteanu model was used to describe the bitumen behavior during binder aging, as reflected in the rheological study results. Ultimately, this study revealed that synthetic wax influences the rheological properties of PMB 45/80-65 polymer bitumen. Specifically, it mitigated the stiffness reduction in modified bitumen caused by polymer degradation during aging at an amount less than 2.5% of synthetic wax.

## 1. Introduction

The ongoing advancement in road construction is crucial for augmenting the technologies used in the creation of road pavements and developing new methodologies. The principal idea involves the production of asphalt mixtures at reduced temperatures during their manufacturing and application. These mixtures are noted for their energy efficiency and lower greenhouse gas emissions, thus promoting environmental sustainability. To attain this goal, it is essential to use a low-viscosity binder produced through a method other than heat application. Consequently, various asphalt additives are utilized to reduce its viscosity. Simultaneously, these additives are anticipated to positively affect the standard and rheological properties of asphalt [1,2,3]. The application of Warm Mix Asphalt (WMA) [4,5,6] or Half-Warm Mix Asphalt (HWMA) technology [7,8,9] facilitates the production of asphalt mixtures. A significant distinction of these technologies is that they operate at markedly lower temperatures than conventional methods, which generally range from 160 °C to 180 °C. Conversely, the technologies mentioned operate at temperatures as low as 60 °C, resulting in a considerable reduction in the necessary thermal energy. The most common additives include synthetic wax [10,11,12], chemicals [13,14,15], and combinations of these additives [16,17].

An alternative method for diminishing the viscosity of asphalt entails the utilization of zeolite [18,19,20] or water [21,22,23] via the process of foaming. Notably, the most substantial decrease in the technological temperatures of the asphalt mixture can be accomplished by foaming the binder with water [23].

At present, the most frequently employed additives are those with low viscosity for the modification of conventional asphalt, which is chiefly applied in the construction of asphalt pavements with low traffic demands [13,16]. Nonetheless, the imperative to protect the environment necessitates the adoption of these measures in polymer-modified bitumen designed for highway paving and road construction, which are anticipated to conform to heightened standards. Significantly, emphasis is placed on ensuring the durability of asphalt pavements, which is linked to guaranteeing the resistance of the asphalt binder to the aging process [24]. During the production of the asphalt mixture and its utilization in pavement, transformations occur within the binder, referred to as technological aging (short-term aging) [25] and operational aging (long-term aging) [26].

During the technological aging process, asphalt is subjected to elevated temperatures reaching up to 180 °C, contingent upon the specific type of asphalt mix produced. Conversely, during the operational phase of asphalt pavements, asphalt undergoes exposure to solar radiation, atmospheric oxygen, and precipitation. These interacting factors induce detrimental alterations in its properties [27,28,29]. The aging of asphalt is characterized by the manifestation of physical and/or chemical transformations that culminate in the diminution of its performance characteristics [30,31]. Generally, the physical and chemical changes associated with asphalt aging predominantly involve the oxidation reactions of its constituents. The outcome is an augmentation in the stiffness and brittleness of the asphalt, accompanied by a reduction in its adhesion to the aggregate. The deteriorating quality of the asphalt mix integrated within the pavement layers precipitates cracking, which predominantly occurs in the wearing course. This process is attributed to the oxidation of asphalt constituents by atmospheric oxygen [32,33]. The oxidation process of asphalt and the consequent increase in its polarity, on one hand, enhance the compatibility between asphalt and SBS. Conversely, it leads to the degradation of temperature-sensitive SBS monomers. Consequently, the resultant increase in homogeneity compromises the viscoelastic properties of modified bitumen. Therefore, one proposed method is the application of synthetic wax, as discussed in the paper. Reducing the asphalt mixing temperature will mitigate the oxidation level and thereby decrease the rate of SBS degradation [6,15,22].

Consequently, when incorporating asphalt additives, it is imperative to evaluate their potential impact on the aging process of the binder and their influence on the rheological properties of the binder. In the proposed experiment, polymer-modified bitumen PMB 45/80-65, augmented with synthetic wax F-T, was employed to examine the influence of the aging process on the resultant changes in the rheological properties of the binder.

Recent scholarly work identifies several unresolved issues in the realm of asphalt modification, with particular emphasis on the impact of synthetic wax on the aging behavior of polymer-modified binders. Despite the prevalent use of synthetic wax additives to decrease viscosity and permit lower production temperatures, their long-term effects on the oxidative aging of modified binders remain insufficiently explored. Current studies offer inconsistent findings concerning the influence of synthetic waxes, such as Fischer–Tropsch wax, on the aging-induced stiffening, embrittlement, and durability of polymer-modified bitumen. This ambiguity underscores a significant research gap necessitating a comprehensive and systematic evaluation of these additives under extended operational conditions. Accordingly, further experimental inquiries are imperative to elucidate these effects and address existing knowledge deficiencies. The originality and research deficiency of this study primarily stem from the limited acknowledgment of the advantageous impact exerted by the incorporation of Sasobit synthetic wax on the aging of polymer bitumens (PMBs). While the general effects of viscosity reduction in asphalt through synthetic waxes are extensively explored within the scientific literature [6], the specific influence of Sasobit on rheological properties and the aging resistance of PMB, particularly concerning the potential for lowering the technological aging process temperature, remains inadequately researched and only partially addressed [22]. Thus, this investigation addresses this gap by systematically examining the modification of PMB 45/80-65 polymer bitumen with Sasobit wax, with a particular focus on the environmental and functional advantages afforded by reduced asphalt aging temperatures. The findings presented herein constitute an innovative contribution to the advancement of knowledge concerning the enhancement of durability and sustainability in road surfaces utilizing polymer-modified bitumen.

## 2. Materials and Methods

### 2.1. Tested Materials

This study used PMB 45/80-65 bitumen, which is widely used in Central and Eastern European countries for the performance of mineral–asphalt mixtures intended for the wearing and binder layers of road pavement structures. The use of this type of asphalt should ensure the resistance of the pavement to the formation of permanent deformations [34], which are loaded by vehicle traffic 2.5 ÷10^6^ < ESAL_100kN_ < 7.3 ÷ 10^6^ (ESAL—equivalent single-axle load) [34]. Table 1 shows the basic values of this binder.

Synthetic wax F-T (SW) was used as an additive to PMB 45/80-65 bitumen, which is solid in the form of very fine granules. Synthetic wax F-T significantly reduces the viscosity of the bitumen. In addition, they provide a high level of adhesion of asphalt to aggregate, which ensures adequate the durability of asphalt pavement. The properties of SW are summarized in Table 2.

### 2.2. Sample Preparation

The procedure for preparing the test samples entailed heating PMB 45/80-65 bitumen to a temperature range between 80 and 90 °C above the softening temperature of the bitumen binder, as specified in EN 12594. Subsequently, 1200 g aliquots of PMB 45/80-65 bitumen were allocated for each specified amount of synthetic wax introduced. This quantity sufficed for the comprehensive testing of asphalt binders with a specified SW content across varying stages: prior to aging, subsequent to RTFOT aging, and following PAV. The specimens were then placed back in the dryer, where the preset temperature was upheld for a duration of 30 min. Subsequently, the procedure involved homogenizing the reference asphalt with a predetermined quantity of SW by means of an electric mixer equipped with variable speed control. This mixing operation was executed over a period of 3 min at a controlled temperature and a rotational speed of 300 rpm. The resultant asphalt binder samples, enhanced by the integration of SW, underwent a quality assessment conducted in conformity with EN 12594:2014 [36] and based on [37].

### 2.3. Experimental Program

PMB 45/80-65 bitumen is modified SW synthetic wax added in amounts ranging from 1.5% to 3.5% in 0.5% increments. The experiment plan was presented in the table below (Table 3).

The analysis demonstrates that all binder scenarios, encompassing the aging stage and test method applied, comprised 12 distinct binder states (4 wax dosages × 3 aging conditions). Each was examined with a number of samples adequate to meet standard precision requirements.

### 2.4. Methods and Modeling Techniques

The initial phase of the research involved evaluating the colloidal state of asphalt through

- The penetration index (PI);
- The yield range (PR) of bitumen.

In the second stage, bitumens’ properties regarding its linear and nonlinear viscoelastic range were studied. These included

- Creep stiffness S (MPa) and the m-value parameter in the BBR test using a bending rheometer;
- Zero shear viscosity η_0_;
- Dynamic modulus |G*|;
- Phase shift angle δ;
- Irreversible creep compliance J_nr3.2_;
- Elastic recovery R_3.2_.

All the parameters studied were determined in three stages:

- Before aging;
- After short-term aging by the RTFOT method (Rolling Thin Film Oven Test) according to EN 12607-1:2014 [38];
- After long-term aging by PAV (Pressure Aging Vessel) according to EN 14769:2023 [39].

In addition, this study included the analysis of photographs of bituminous binders with the addition of viscosity-reducing agents taken with an epi-fluorescence microscope.

#### 2.4.1. Penetration Index and Yield Range

Bitumen PMB 45/80-65 incorporated a modified SW synthetic wax at concentrations ranging from 1.5% to 3.5%, with increments of 0.5%. The determination of the penetration index (PI) values is carried out as specified in Equation (1), considering its consistency at ambient temperature by measuring the penetration at 25 °C (Pen, EN 1426:2015-08 [40]) and the softening point (TR&B, EN 1427:2015-08 [41]). For this analysis, the formula outlined in EN 12591:2009 [42] was employed to compute PI:

(1) $$PI=\frac{20{\cdot T}_{R\&B}+500\cdot \log\left(Pen\right)-1952}{T_{R\&B}+50\cdot \log\left(Pen\right)-120}$$

The penetration index characterizes changes in the consistency of a binder as a function of temperature changes and is dimensionless (-). It allows for the evaluation of its suitability, especially in countries with temperate climates characterized by a large temperature gradient due to the rise in high temperatures in summer and low temperatures in winter.

The yield range (PR) of the binder, which depends on its softening point (T_R&B_) and breaking point (T_Fraass_, EN 12593:2015 [43]), has been determined by Equation (2) in accordance with the requirements of the following formula:

(2) $$PR=T_{R\&B}-T_{Fraass}\;({}^{\circ}C)$$

where

T_R&B_—softening temperature according to the “Ring and Ball” method (°C);
T_Fraass_—Fraass breaking point (°C).

#### 2.4.2. Rutting Potential G*/sin(δ) in a DSR

G*/sin(δ) measures the stiffness of the binder at high summer temperatures. Obtaining a high value of the G* modulus and a low value of the phase angle δ is advisable to minimize permanent deformations. Such a relationship allows for a highly elastic response of the binder. The values depend largely on the temperature and frequency of the applied load. The frequency value of 1.96 Hz at which the G*/sin(δ) test was performed corresponded to approximately 80 km/h vehicle speed, as per the provisions of the SUPERPAVE program (SHRP) [37]. This study used a measuring system of parallel plates with a diameter of 25 mm and a gap of 1 mm. The measurement temperatures were 50 °C, 60 °C, and 70 °C. In Poland, the recommended value of G*/sin(δ) is ≥1.0 kPa for unaged bitumen at the maximum pavement temperature.

#### 2.4.3. Bending Beam Rheometer Test

Considering that the Fraass breaking point test is a test that does not reflect the actual temperature of transition of asphalt to the glassy state, the low-temperature properties of asphalt binders before and after the long-term aging process of PAV were additionally determined according to the methodology adopted by Superpave (USA). The determination of flexural creep stiffness was performed in a bending beam rheometer (BBR) according to EN 14771:2012 [44]. The measurement was carried out for four temperature values: −10 °C, −16 °C, −22 °C, and −28 °C. The BBR test was designed to determine the degree of deflection (creep) of an asphalt beam with standard dimensions of 125 mm × 12.5 mm × 6.25 mm under an automatically applied load of 980 ± 50 mN for 240 s. During the test, the beam deflection was continuously recorded to calculate the creep stiffness modulus (S_m_) after the loading time of 60 s and the change in creep stiffness (m). As a result, the minimum critical temperature T_crit_ was determined using the BBR test at Sm = 300 MPa and T_crit_ at m = 0.3.

#### 2.4.4. The Christensen Anderson Marasteanu (CAM) Model

The Christensen Anderson Marasteanu (CAM) model [45,46] described by Equation (3) was used to describe the behavior of asphalt binders with SW synthetic wax added during the aging process. The CAM model is an attempt to more accurately describe the stiffness of both unmodified and modified bitumen by the following relationship [45,46,47] (3):

(3) $$\left|G^{*}\right|=G_{g}\cdot {\left[1+{\left(\frac{f_{c}}{f}\right)}^{v}\right]}^{-\frac{w}{v}}$$

where $G^{*}$—complex shear, $G_{g}$—glassy modulus, $f_{c}$—the frequency at the cross point (Hz), f—measurement frequency, and *w* and *v*—experimental curve fitting parameters of the CAM model.

The CAM model defines the rheological index *R* as follows:

(4) $$R=\frac{log2}{k}$$

The obtained test results were subjected to a static analysis to determine their reliability and to determine the significance of the effect of the synthetic wax F-T on the properties of asphalt PMB 45/80-65 using an ANOVA (analysis of variance) [48,49]. To ensure the reliability of the results obtained for the properties of asphalt PMB 45/80-65 modified with SW synthetic wax, the determination of each parameter was carried out on nine samples [49]. The results of this study were analyzed using Statistica software [50] to ensure their reliability and to identify significant relationships between the binder parameters tested and the amount of synthetic wax used.

## 3. Results

### 3.1. Influence of Synthetic Wax on the Penetration Index PI and Temperature Range of Plasticity PR

The PI is a measure of the thermal sensitivity of asphalt. The lower its value, the faster the binder changes its consistency with temperature changes (it has greater thermal sensitivity) [51]. Meanwhile, PR is the temperature range in which an asphalt binder retains its viscoelastic properties, i.e., it is in a viscoelastic state [52]. A good asphalt should have low stiffness at low temperature and low elastic transition temperature, and high stiffness at high pavement service temperatures [52]. The viscous transition temperature, on the other hand, should be above the highest temperature induced by solar radiation in the pavement.

It should also be noted that the penetration, softening temperature, and fracture temperature tests were performed on the same binder sample. Therefore, it is likely that there is a correlation between these parameters caused by the existence of the same group composition of asphalt. Therefore, the values of the PI and PR parameters are declared values, the purpose of which was to illustrate the trend of the change in viscoelastic characteristics occurring in the samples.

**Figure 1:**
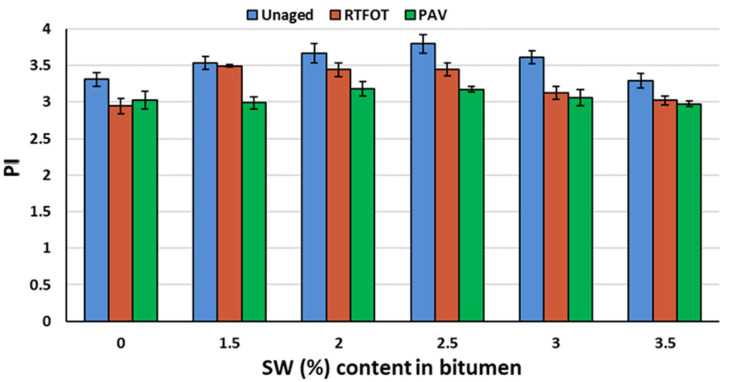
PI of PMB 45/80-65 bitumen as a function of WS (%) before and after RTFOT and PAV aging.

**Figure 2:**
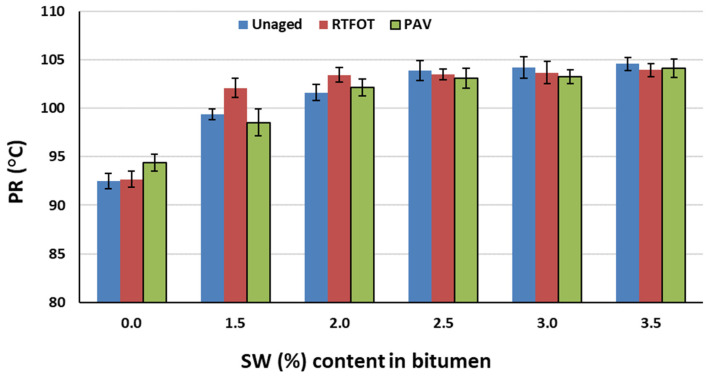
PR dependence of PMB 45/80-65 bitumen on SW (%) before and after RTFOT and PAV aging.

The changes in the values of calculated PI and PR parameters of PMB 45/80-65 bitumen with the addition of SW synthetic wax before aging and after RTFOT and PAV aging are shown in Figure 1 and Figure 2.

From the analysis of the test results shown in Figure 1 and Figure 2, it can be concluded that the addition of up to 2.5% synthetic wax to PMB 45/80-65 bitumen contributes to an increase in the penetration index value. Above this amount of low wax in the binder, the colloidal stability of the asphalt is likely to be violated and the PI value will decrease. Regardless of the type and amount of viscosity modifier in all of the asphalts tested, the aging process caused a decrease in the penetration index value and thus an increase in the thermal sensitivity of the binder. Nevertheless, all the asphaltenes tested have a PI above +2, which means that they are gel-type asphaltenes. They are characterized by low temperature sensitivity. The transition from the solid to liquid states occurs slowly with increasing temperature (they have a large viscoelastic range) [52]. Such binders retain viscous properties at low temperatures and short loading times and do not flow at positive temperatures and long loading times. A minor reduction in the penetration index following the aging process may be attributable to the degradation of SBS [52]. However, this effect was likely mitigated by the presence of synthetic wax.

Analyzing the test results shown in Figure 1 and Figure 2, there is a significant increase in the temperature range of plasticity as the amount of SW in the asphalt binder increases (up to 13 °C for its 3.5% content). The aging process did not induce significant changes with respect to the properties of the bitumen binder before aging.

### 3.2. Bending Beam Rheometer (BBR) Creep Stiffness of PMB 45/80-65 Bitumen with Synthetic Wax Additive

The asphalt stiffness measurement using the BBR test was used to evaluate the stiffness of the asphalt binder sample (determination of stiffness modulus S) and to study the phenomenon of creep under static loading and recovery after unloading (determination of m parameter). In order to maximize the informative value of the evaluation of both the low-temperature properties and the rheological properties of the asphalt binders to be determined, the experimental design was reduced to testing PMB 45/80-65 asphalt with SW contents of 1.5%, 2.5%, and 3.5%. This was possible on the basis of previous studies where small differences in results were obtained between individual determinations for a given parameter.

**Figure 3:**
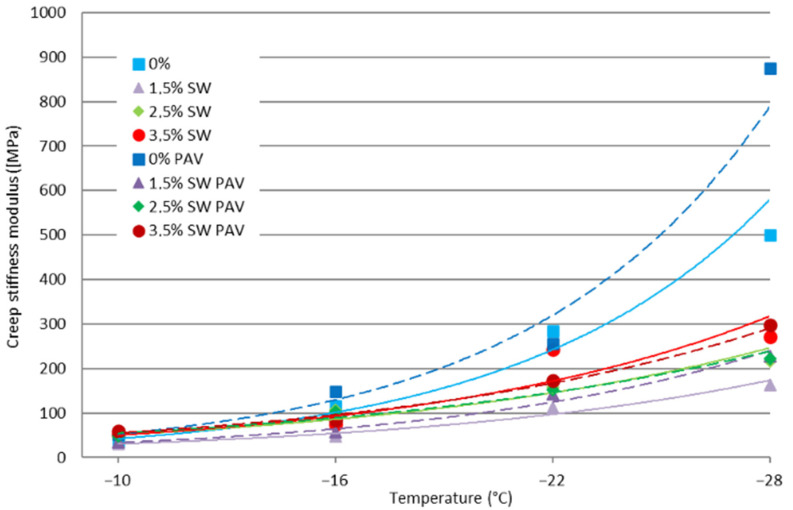
Stiffness modulus S of PMB 45/80-65 bitumen as a function of SW content before and after PAV aging.

**Figure 4:**
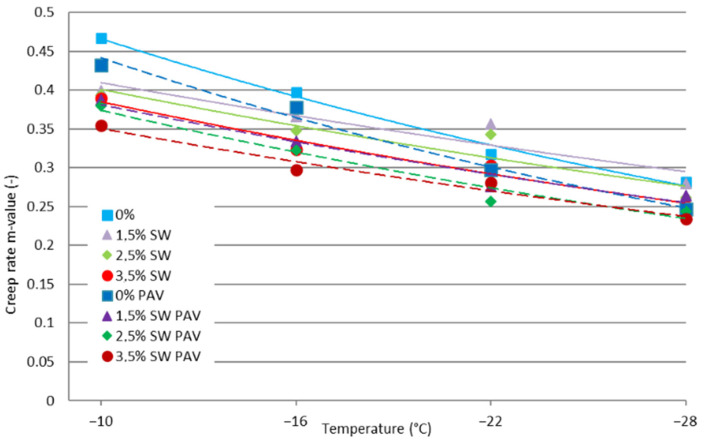
Values of the m parameter of SW synthetic wax bitumen before and after PAV.

The results of the low-temperature rheological properties of asphalt binders containing SW before and after long-term aging of PAV are shown in Figure 3 and Figure 4.

The use of synthetic wax in PMB 45/80-65 bitumen, regardless of the test temperature, resulted in a significant decrease in the value of the stiffness modulus S (the greatest for its 1.5% content in the reference bitumen). The PAV process contributed to an additional increase in the value of the tested parameter. The greatest increase in stiffness modulus S after PAV aging was recorded for PMB 45/80-65 bitumen. The higher the content of synthetic wax in the bitumen binder, the smaller the changes in the value of the parameter during aging.

Based on the results of the study of the change in stiffness (parameter m) shown in Figure 4, it can be concluded that as the test temperature decreases, the rate of change in the stiffness of the binder during loading decreases.

It can be concluded that the use of SW in PMB 45/80-65 bitumen, regardless of its amount and testing temperature, caused a decrease in the value of the parameter m. The higher the content of SW in the asphalt binder, the lower the value of the tested parameter. The process of long-term aging of PAV in all variants of the addition of synthetic wax to polymer-modified bitumen contributed to an additional decrease in the value of the parameter m. This is not beneficial, because with a lower value of the parameter m, such binders will be characterized by more effective stress relaxation, which will contribute to the formation of low-temperature cracks

**Figure 5:**
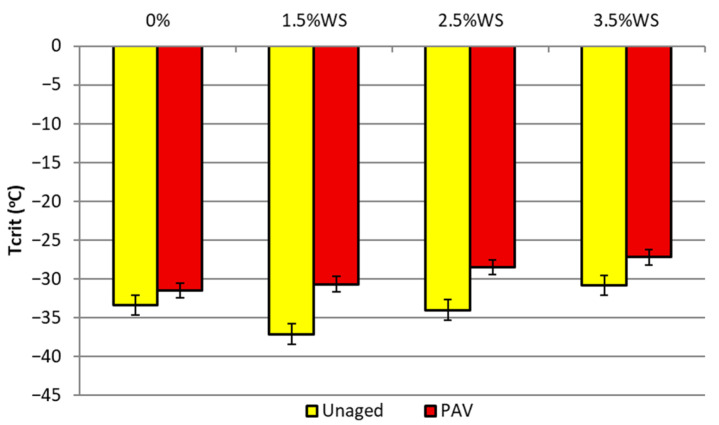
Critical temperature for PMB 45/80 bitumen with SW additive before and after PAV.

Critical temperatures for PMB 45/80-65 bitumen with the addition of SW synthetic wax before and after long-term aging of PAV are shown in Figure 5.

From the results of the binder tests in the BBR rheometer shown in Figure 5, it can be concluded that PMB 45/80-65 bitumen with 1.5% and 2.5% synthetic wax content contributes to a decrease in the value of the critical temperature compared to the reference bitumen. With an increase in the amount of this additive in the binder, an unfavorable increase in the value of the tested parameter can also be observed.

### 3.3. Binder Rheological Properties in Terms of Linear (LVE) and Nonlinear (N-LVE) Viscoelasticity

#### 3.3.1. Effect of Aging on Zero Shear Viscosity

Zero shear viscosity (η_0_) is one of the parameters that can be used to evaluate the resistance to permanent deformation of mineral–asphalt mixtures. In an asphalt binder, which is a thermoplastic material, η_0_ depends on the test temperature and the additives used to modify it [53]. The higher the value of zero shear viscosity, the better the strength properties of bitumen binders at high road pavement operating temperatures [54].

In this dissertation, the zero shear viscosity was determined using the creep method in accordance with PKN-CEN/TS 15325. It is an extrapolated value in relation to the shear rate or for frequencies going to zero. It should also be noted here that it can be difficult, even impossible, to determine the zero shear viscosity of bitumen as well as the level of viscosity independent of loading time. This is especially likely for liquids such as highly modified bitumen and others with pronounced yield stress. Therefore, the zero shear viscosity value of the bitumen was assigned to the viscosity read for low shear velocity (LSV), which characterizes the material in the range when the shear velocity dependence of viscosity was not revealed. As the value of the frequency for which the plateau level (η_0_) was determined, the value ω = 0.005 Hz was set, that is, the shear rate γ = 0.0032 s^−1^ [55]. The viscosity value η_0_ was read using a modified Cross model and referred to as the Cross/Sybilski model [56] using Equation (5):

(5) $$\eta^{*}=\frac{\eta_{0}}{1+K\omega^{m}}$$

where

K ∈ (0;1), m ∈(0;70)—experimental parameters specific to the fluid;
$\eta_{0}$—zero shear viscosity (parameter estimated by least squares).

**Figure 6:**
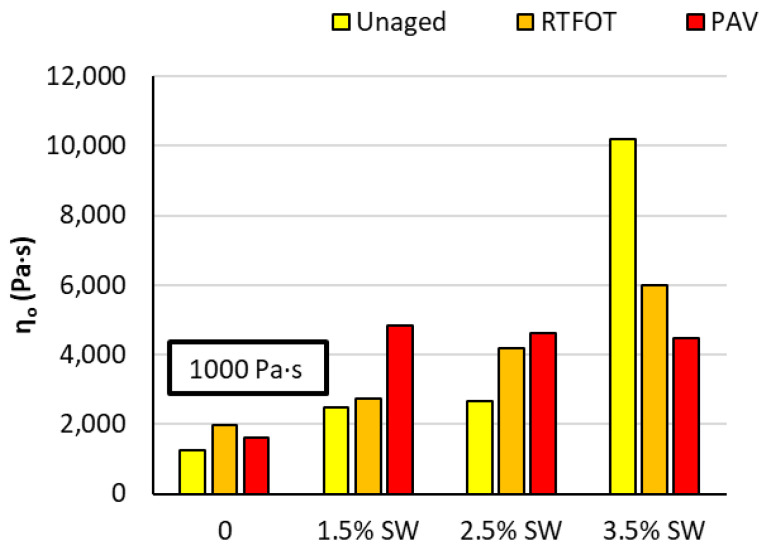
Zero shear viscosity η_0_ at 60 °C before and after RTFOT and PAV aging for PMB 45/80-65 with SW synthetic wax additive.

The aging process induces changes in both the bitumen and the polymer [52]. There is a decrease in the molecular weight of the bitumen, so changes in zero shear viscosity are expected first. The results of the viscosity η_0_ at 60 °C before and after the short- and long-term aging process for 45/80-65 bitumen with SW synthetic wax are shown in Figure 6.

The critical value of the parameter η_0_ for unaged bitumen is 1000 Pas [44,55]. Below this value, the occurrence of the third stage of the rutting process (a stage with no change in volume but with a significant uncontrolled increase in deformation) is very often observed in mineral asphalt mixtures [55]. The results of the tests show that all variants of modification before and after short-term (RTFOT) and long-term aging (PAV) obtained viscosity values η_0_ > 1000 Pas. Therefore, the analyzed asphalt binders will contribute to the reduction in rutting. The higher the value of zero shear viscosity, the lower the rate of rutting in the mineral–asphalt mixture. Similar conclusions were reached by the authors of [55].

In the case of PMB 45/80-65 bitumen modified with SW synthetic wax, an increase in the amount of this additive results in an increase in the value of the η_0_ parameter both before and after the short-term and long-term aging process. This means that the rate of growth of permanent deformation in the bitumen is significantly reduced, which reduces the probability of the appearance of functional ruts in the asphalt pavement layer. Nevertheless, a decrease in viscosity η_0_ was observed after RTFOT and PAV aging at 3.5% synthetic wax content. This phenomenon may be related to the fact that the source bitumen used in the study is a binder with a significant polymer content (PMB 45/80-65). The polymer in the bitumen may have degraded during the aging process, resulting in a decrease in the cross-linking ability of the binder structure, despite the lower temperature of the RTFOT aging simulation. The degradation phenomenon of highly polymer modified bitumen has been observed in many works [56,57]. An increase in the number of synthetic wax crystallites in the aged binder causes an increase in the molecular weight of the bitumen, which consequently contributes to a rapid violation of its structure under shear stress.

#### 3.3.2. Viscoelastic Properties of SW-Added Binder Based on Black’s Curves

Black’s curves are an important way of presenting the effect of aging on the properties of PMB 45/80-65 bitumen with different modifiers in WMA technology. They provide a graphical representation of the dependence of the dynamic modulus |G*| on the phase shift angle δ. They also illustrate the rheological character of the bitumen and allow for a graphical comparison of different modification variants of bitumen binders. In addition, this type of presentation allows for conclusions to be drawn about the viscoelasticity of SW-modified PMB 45/80-65.

**Figure 7:**
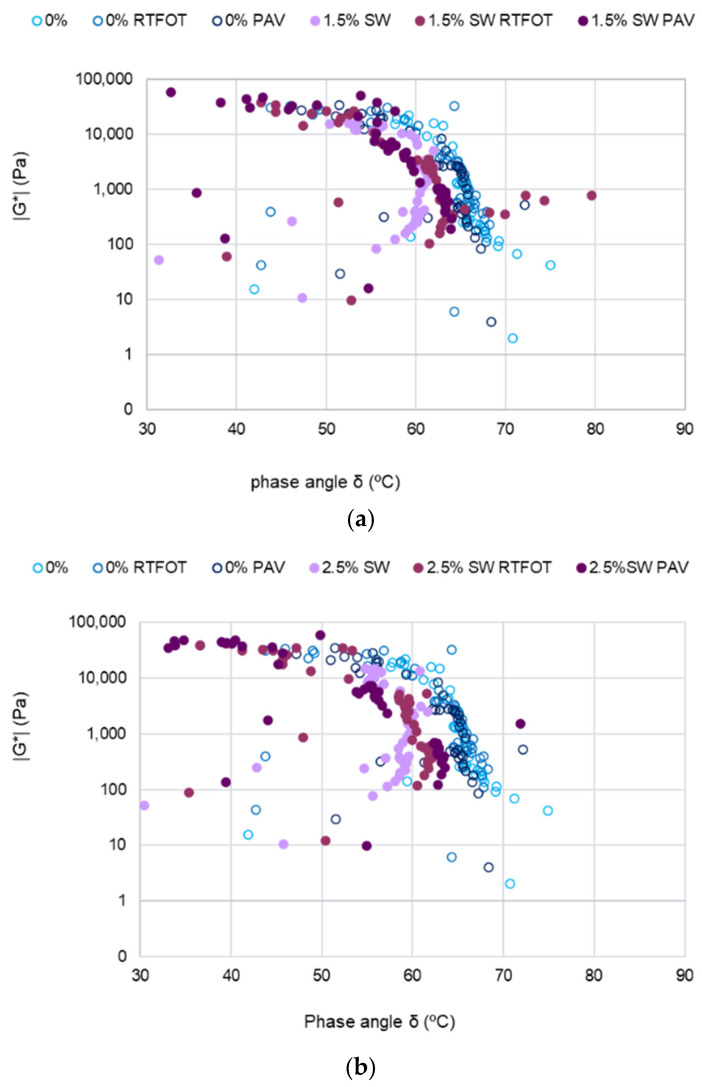

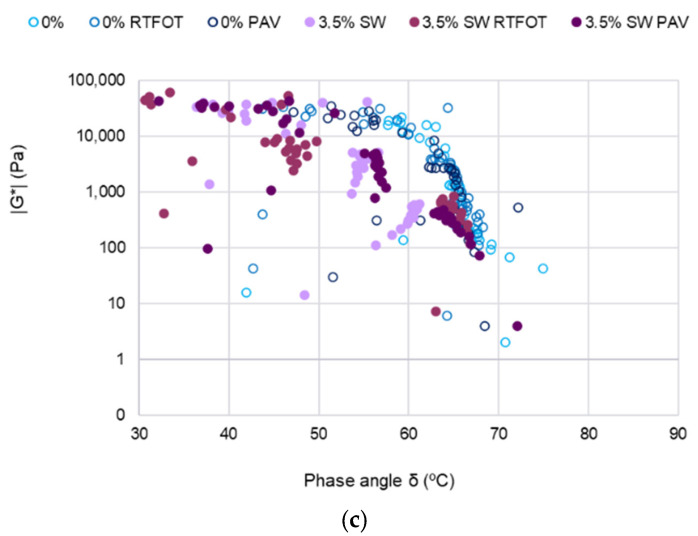
Black’s curves for PMB 45/80-65 bitumen before and after RTFOT and RFTOT+PAV: (**a**) PMB+1.5%SW; (**b**) PMB+2.5%SW; (**c**) PMB+3.5%SW.

Black’s curves were generated based on the results of dynamic tests of the binder at 40 °C, 60 °C, and 80 °C in the frequency range of 0.01 Hz to 10 Hz. Graphs of Black’s curves of binders with SW additive before and after RTFOT and RTFOT+PAV aging, together with graphs for PMB 45/80-65 bitumen, are shown in Figure 7.

Based on the analysis of the test results (Figure 7), it can be concluded that an increase in the amount of SW synthetic wax in the PMB 45/80-65 binder causes a decrease in the phase shift angle δ. This suggests that the elastic part G′ of the dynamic modulus will dominate in the binder. For unaged bituminous binders, both the synthetic wax content and the polymer content will contribute to an increase in the value of the dynamic modulus |G*|. In addition, in the case of polymer-modified bitumens with the addition of SW, a characteristic curvature is observed, which indicates favorable rheological properties of the asphalt binder from the point of view of its use. On this basis, it can also be concluded that at high test temperatures, the level of elastic recovery will increase. However, when each type of asphalt binder with SW additive was considered separately, it was found that the phase shift angle after aging was smaller than before aging. This probable effect of partial polymer degradation may affect the compatibility of the composite composed of PMB 45/80-65 bitumen and a large number of synthetic wax crystallites. The aging process causes an increase in the phase shift angle of PMB 45/80-65 bitumen with SW to a level close to that of the reference bitumen. The difference in phase shift angle between the reference bitumen and the aged PAV with the addition of 3.5% synthetic wax is about 5° (Figure 7c). Therefore, the effectiveness of the modification of the PMB 45/80-65 bitumen with the addition of the SW modifier is compensated by the aging process. In addition, the results of the determination of the dynamic modulus |G*| of PMB 45/80-65 + 3.5% SW bitumen, after the PAV long-term aging process, lie along a vertical line. The constant value of the phase shift angle δ indicates an increase in the proportion of the viscous part G″ with a constant level of the elastic part G″ in the results of the determination of the dynamic modulus |G*|. This finding corroborates earlier speculations that an augmentation in the quantity of synthetic wax crystallites and asphaltenes can precipitate instability in the polymer-modified asphalt mixture [58,59].

#### 3.3.3. Effect of Binder Aging on Cyclic Creep and Recovery with SW Additive

The study of the properties of high-temperature asphalt binders was further supplemented by the Multiple Stress Creep Recovery (MSCR) test. The essence of this test was to measure the properties of the bitumen binder at the highest expected pavement service temperature and at any chosen reference temperature. The test results determine the influence of the binder on the resistance of the asphalt mix to permanent deformation (rutting) and, in the case of PMB, assess the degree and effectiveness of polymer modification (material elasticity) [32].

The test was performed at 40 °C and 60 °C on a suitably configured DSR dynamic shear rheometer. In accordance with the standards AASHTO TP 70 [60] and EN 16659 [61], the apparatus used a parallel plate arrangement with a diameter of 2.5 cm and a gap of 1 mm. During the MSCR test, the following mechanisms were investigated:

- The creep mechanism of the binder specimen—during 1 s stress;
- The recovery mechanism of the binder specimen—during a 9 s annealing period (after stress relief) [32].

Ten cycles of creep and relaxation were performed at a shear stress of 0.1 kPa, followed by another 10 cycles at a shear stress of 3.2 kPa. As a result of the cyclic creep stress MSCR test performed, two pairs of results were obtained:

- Irreversible creep compliance, denoted as J_nr3.2_—the index of resistance of the asphalt binder to rutting, expressed in [kPa^−1^], determined for two stress levels: 0.1 kPa and 3.2 kPa;
- Elastic recovery R_3.2_, the elasticity index of the binder at a given temperature, expressed in [%], determined for two stress levels: 0.1 kPa and 3.2 kPa.

**Figure 8:**
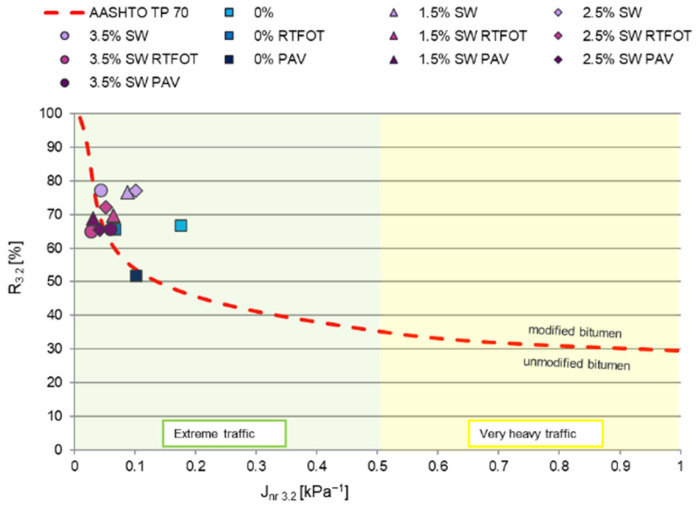
Presentation of MSCR test results: recurrence of R as a function of J_nr_ at a shear stress of 3.2 kPa at 60 °C of PMB 45/80-65 bitumen with SW additive before and after RTFOT and PAV.

**Figure 9:**
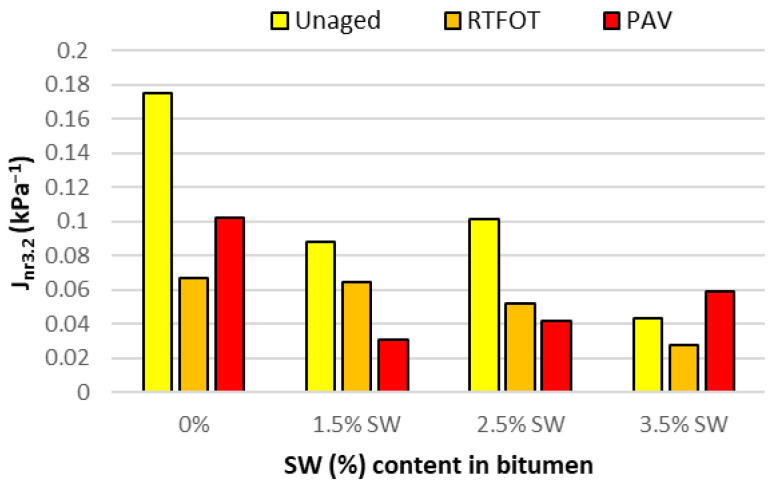
Creep compliance J_nr3.2_ (kPa^−1^) of asphalt binders with SW and before and after RTFOT and PAV at 60 °C at a shear stress of 3.2 kPa.

**Figure 10:**
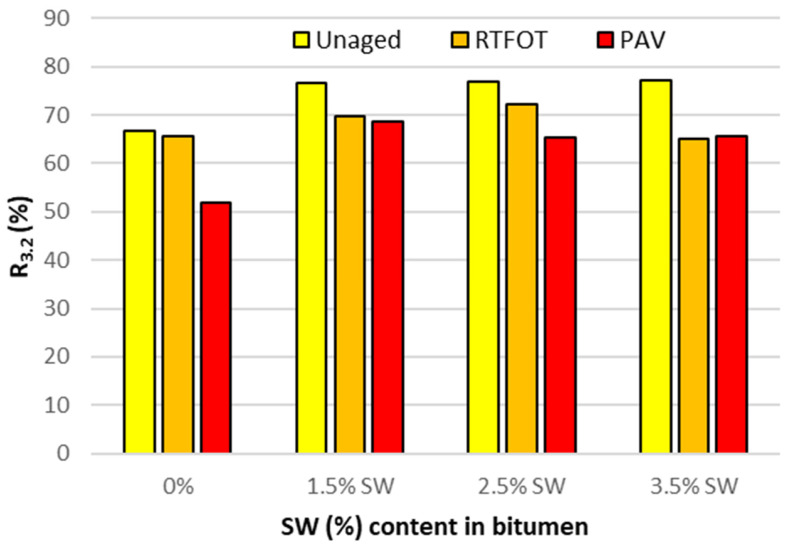
Elastic recovery R (%) of bitumen with SW before and after RTFOT and PAV at 60 °C at a shear stress of 3.2 kPa.

The results of the J_nr_ and R parameters obtained at a stress of 3.2 kPa (shown in Figure 8, Figure 9 and Figure 10) are key to the classification of the asphalt binder (according to Superpave). The value of J_nr3.2_ at 3.2 kPa (J_nr3.2_) determines the resistance of the binder to permanent deformation—the lower the J_nr3.2_ value, the greater the resistance to rutting. It is also believed that the parameter J_nr_ at 3.2 kPa is a better characterization of bitumen properties in terms of predicting susceptibility to permanent deformation than the |G*|/sinδ parameter or the softening temperature [62]. In contrast, a recurrence score of R at 3.2 kPa (R_3.2_) indicates the effectiveness of the binder modification. According to D’Angele [63], the MSCR test has a significant advantage over the elastic recurrence or ductility test in that it provides information not only on whether the bitumen is modified with a polymer but also on its structure, bond strength, type of modification, and stability.

A red dashed line separating modified from unmodified bitumen (i.e., binders meeting the requirements for modified bitumen in the R_3.2_ recurrence range correlated with the ranges of J_nr3.2_ values) was also plotted on the graphs, showing the dependence of the percentage elastic elongation on the irreversible part of the modulus of elasticity (Figure 8) [64]. This line was determined experimentally by American researchers and can be calculated using the equation y = 29.371·(J_nr3.2_)^−0.2633^ [65].

The addition of SW synthetic wax to PMB 45/80-65 bitumen contributed to an increase in the recurrence value R_3.2_ relative to the reference bitumen, with a concomitant decrease in the creep compliance J_nr_ at 3.2 kPa. This indicates that polymer-modified bitumen with the addition of synthetic wax becomes a more elastic material with higher stiffness. This confirms the hypothesis that PMB 45/80-65 bitumen with the addition of SW wax increases the predominance of the elastic part of the dynamic modulus. The lower value of the irreversible modulus of elasticity J_nr_ at 3.2 kPa of asphalt binders with the addition of the modifying agent SW relative to the base bitumen, on the other hand, indicates that there will be greater resistance to permanent deformation of the mineral–asphalt mixture with such a binder. On this basis, it can also be concluded that the reference bitumen with the addition of the synthetic wax SW would ideally be suitable for pavement layers made of ACWMS (high modulus asphalt concrete) in WMA technology.

In order to verify the degree of modification of the tested binders, Figure 8 also shows the curve marked with a red dashed line [65]. Although the asphalt binders with the addition of synthetic wax have different viscoelastic characteristics, almost all of the tested modification variants of PMB 45/80-65 bitumen meet Superpave’s modification quality requirements (they take values above the curve). This shows that, according to the AASHTO TP 70 standard [60], they meet the requirements for modified bitumens in the R_3.2_ recurrence range correlated with the J_nr3.2_ value intervals.

In addition, all the binders analyzed with the addition of viscosity modifiers, irrespective of the aging stage, achieved J_nr3.2_ < 0.5 kPa^−1^, i.e., they meet the J_nr3.2_ requirements for extreme traffic—“E” (can be used for roads with both more than 30 million axles and speeds below 20 km/h) according to the PG+ binder functional test system.

For a more detailed analysis of the values obtained for irreversible creep compliance and percentage recovery, Figure 9 and Figure 10 show the effect of the addition of SW synthetic wax on the J_nr_ and R parameters of asphalt binders at 60 °C under a shear stress of 3.2 kPa before and after short- and long-term aging.

On the basis of the analysis of the results presented in Figure 9, it can be concluded that the addition of SW synthetic wax to PMB 45/80-65 bitumen, in each of the dosage variants results in a decrease in J_nr3.2_ of the bitumen binder. Depending on the low-viscosity agent used, the short- and long-term aging process has a different effect on the change in the value of the parameter studied. Asphalt binders with SW have a lower value of J_nr3.2_ after RTFOT and PAV aging compared to the base bitumen. This shows that asphalt mixes made with PBM 45/80-65 bitumen with the addition of SW wax have a higher rutting resistance than mixes made with unmodified reference bitumen. This means that after long-term aging, such binders will have a lower resistance to permanent deformation than the reference PBM 45/80-65 bitumen. In addition, D’Angelo’s work [50] showed that doubling the value of J_nr3.2_ also doubles the rut depth.

When analyzing the results of the R_3.2_ recurrence tests shown in Figure 10, it can be seen that the SW-modified bitumen binders obtained higher values for the tested parameter than the reference bitumen. In all cases tested, both the RTFOT and PAV aging processes result in a reduction in the elasticity of the bitumen binder. Nevertheless, the values of parameter R_3.2_ after long-term aging of the bitumen modified with the F-T wax polymer are still higher than those of the base bitumen. This is fully consistent with previous test results and is associated with an increase in the |G*| modulus. It also means that the stress range in which the SW-modified bitumen binder is in the viscoelastic range is extended. On this basis, it can also be concluded that the addition of SW to PMB 45/80-65 can significantly improve the rutting resistance of mineral–asphalt mixes by giving them a more elastic character at 60 °C, which is considered to be the highest pavement temperature in summer.

DSR dynamic shear rheometer tests have shown that as the amount of synthetic wax (SW) added to PMB 45/80-65 bitumen increases, the zero shear viscosity η_0_ increases both before and after short- and long-term aging. In an unaged bitumen binder, the synthetic wax content also contributes to an increase in the dynamic modulus value |G*| and causes a decrease in the phase shift angle δ. This means that the rate of permanent deformation growth in the bitumen is significantly reduced, thereby reducing the likelihood of functional rutting in the asphalt pavement layer.

In addition, it was found that after aging at a high SW synthetic wax content of 3.5% in PMB 45/80-65 bitumen, there is a decrease in viscosity η_0_ and an increase in dynamic modulus |G*| at a constant value of phase shift angle δ. This suggests that the polymer in the bitumen may have degraded during aging, which may affect the compatibility of the composite composed of PMB 45/80-65 modified bitumen and a large amount of synthetic wax crystallites.

MSCR tests performed at 60 °C and 3.2 kPa stress showed that the addition of synthetic wax to PMB 45/80-65 bitumen contributes to an increase in the R_3.2_ recurrence value with a decrease in J_nr3.2_. This means that the polymer-modified bitumen with the addition of the viscosity modifier SW will be a more elastic material with higher stiffness than the reference bitumen. In addition, after aging, asphalt binders with the addition of SW synthetic wax have a lower J_nr3.2_ value and a higher R_3.2_ value than the base bitumen, which will have a beneficial effect on the rutting resistance of mineral asphalt mixtures with such a binder, giving them a more elastic character at 60 °C.

### 3.4. Construction of a CAM Model of a Modified Binder

As a result of the rheological tests carried out on the modified bitumen PMB 45/80-65 with SW, the viscoelastic properties of the binder, such as the dynamic modulus |G*| and the phase shift angle δ, were determined at three temperature values (40 °C, 60 °C, and 80 °C) and in the frequency range from 0.01 to 10 Hz. The obtained dynamic modulus results served as an input set for the estimation of the CAM model parameters. The parameters were calibrated using the nonlinear least squares method. The search for the optimum solution, which should match the experimental data as closely as possible with the data obtained from the model, was carried out by simultaneously applying the Hooke–Jeeves and Quasi–Newton solvers [66]. A temperature of 60 °C was used as the reference temperature for the horizontal shift factor for the leading curve model. This resulted in a set of CAM model parameters: fc, w, and v (Table 3). The value of the glassiness modulus Gg, which was one of the CAM model parameters, was determined in the BBR study at −28 °C. It was assumed that at such a low temperature, the asphalt binder behaves almost perfectly elastic (the phase shift angle is close to 0°) and the values of the stiffness modulus S obtained in the BBR test do not depend on the loading time [67]. Finally, for PMB 45/80-65 bitumen with viscosity modifiers, both before and after RTFOT and PAV aging, the level of R^2^ coefficients of determination obtained is no less than 99%. This demonstrates the high accuracy of the fit of the CAM model curve to the experimental results obtained at 60 °C.

The resulting CAM model fitting parameters, shown in Table 4, were also used to estimate the rheological index R. This is a very useful parameter as it is very sensitive to changes in the stiffness of the bitumen in relation to load time/temperature. Therefore, a small change in the stiffness of the bitumens analyzed, caused by the aging process and chemical changes in the bitumen binder, will result in a noticeable change in the rheological index.

**Figure 11:**
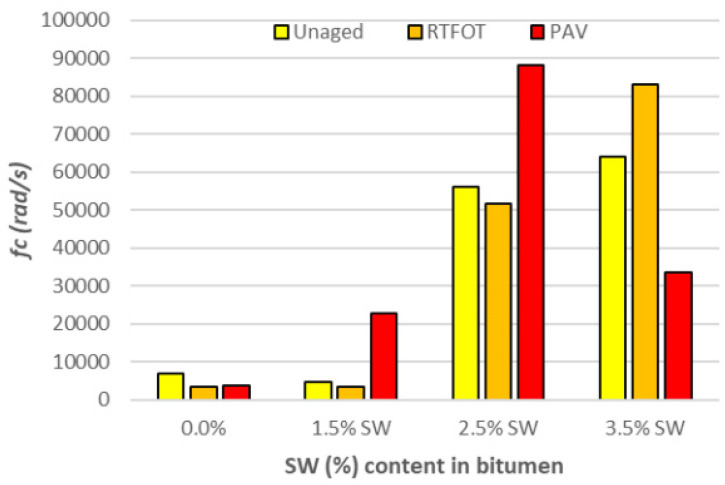
Cross point frequency values (rad/s) f_c_ before and after RTFOT and PAV for PMB 45/80-65 bitumen with SW additive.

**Figure 12:**
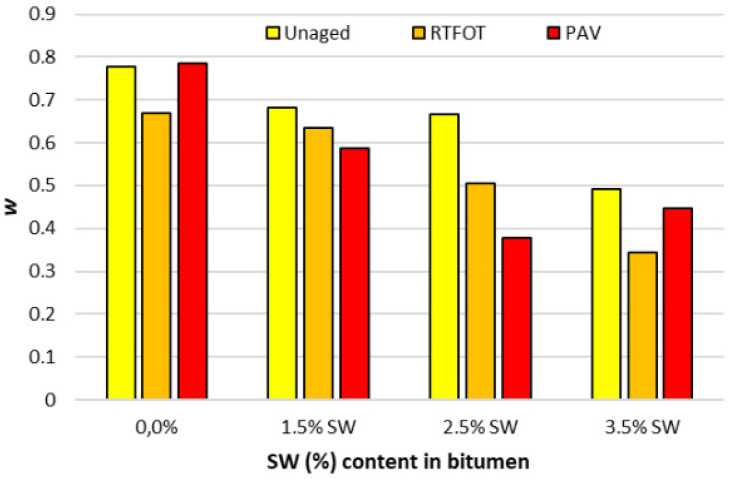
W parameter before and after RTFOT and PAV process for PMB 45/80-65 bitumen with SW additive.

**Figure 13:**
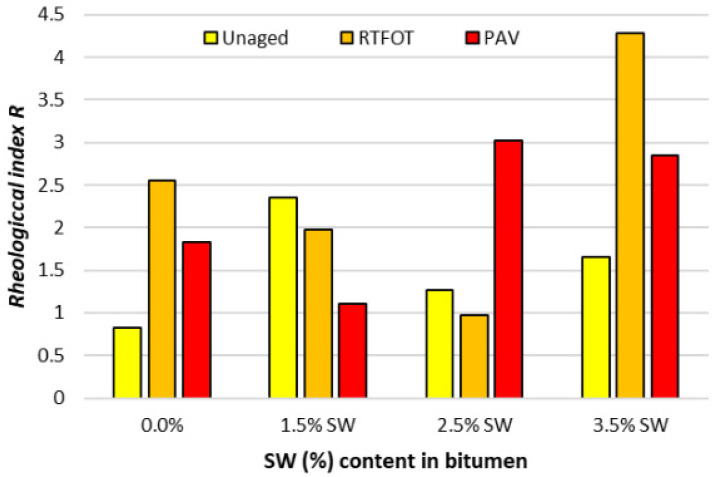
Rheological index R values before and after RTFOT and PAV process for PMB 45/80-65 bitumen with SW additive.

A graphical interpretation of the estimated parameters f_c_ and in the CAM rheological model and the rheological index R calculated according to Formula (4) is shown in Figure 11, Figure 12 and Figure 13.

An analysis of the results of the fc and w parameters shown in Figure 11 and Figure 12 indicates that as the amount of SW synthetic wax additive increases, the bitumen binder becomes a stiff material with a low frequency range over which stress relaxation occurs (the width of the relaxation spectrum decreases). In addition, the lower value of the w parameter compared to the PMB 45/80-65 bitumen with surfactant addition indicates that the rate at which the binder with SW addition reaches viscosity is extremely low.

It can also be seen from the test results (Figure 13) that the rheological index R reaches significantly higher values for the polymer-modified bitumen with the synthetic wax additive than with the surfactant additive. In addition, the level of the rheological index R increased in the bitumen with the addition of SW compared to the reference bitumen after the aging process. This means that the sensitivity of such an asphalt binder to loading time is reduced. This fact also indicates high homogeneity of the composition of polymer-modified bitumen and synthetic wax. The presence of synthetic wax crystallites occurs in the form of “needles” dispersed in the polymer phase. This is also confirmed by previous observations of the spectrum of the asphalt binder, where the presence of synthetic wax crystallites dominates the image of the polymer phase. This also implies that the presence of SW synthetic wax can compensate to a much greater extent than surfactants for the decrease in viscosity of polymer-modified bitumen caused by polymer chain degradation.

**Figure 14:**
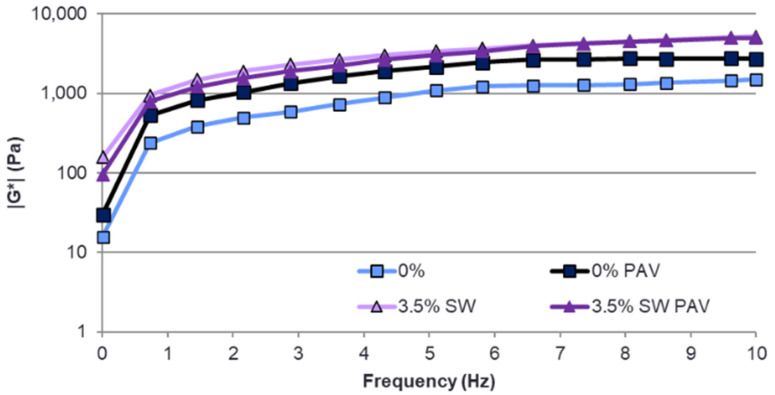
Change in dynamic modulus |G*| as a function of frequency at 60 °C for PMB 45/80-65 and with 3.5% SW synthetic wax added before and after PAV aging.

In order to fully characterize the binder as a visco-elastic material, an analysis was also carried out to evaluate the change in dynamic modulus |G*| as a function of frequency. The results obtained using the CAM model for PMB 45/80-65 bitumen and bituminous binders with 3.5% synthetic wax added before and after PAV aging are shown in Figure 14.

The frequency represents the potential loading time of the pavement by the vehicle axle. Therefore, the low frequency range between 0 and 2 Hz, where asphalt pavements are primarily subjected to the deformation process, is very important. This range represents the time during which the pavement is loaded by vehicles travelling at speeds of up to 60 km/h. The higher the value of the dynamic modulus in this range, the greater the resistance of the asphalt mix to rutting.

From the test results shown in Figure 14, it can be concluded that the addition of synthetic wax to the reference bitumen increases the |G*| value compared to the values obtained for the reference bitumen. For a temperature of 60 °C and a frequency range of 0 to 2 Hz, the sensitivity of the bitumen to loading time for the binder modified with 3.5% synthetic wax is almost 10 times lower than for the reference bitumen PMB 45/80-65. This indicates the likely beneficial effect of such a bitumen on the resistance of asphalt pavements made with polymer-modified bitumens with the addition of SW wax to permanent deformation caused by repeated loading cycles. Here, the presence of synthetic wax crystallites can act as a fine crystalline filler, thereby increasing the dynamic modulus of the bitumen.

The results of the experiment to simulate the aging process of polymer-modified bitumen with viscosity-reducing agents varied according to the shape of the sigmoidal function of the CAM model. This confirms that the Christensen Anderson Marasteanu model is a good mathematical tool for describing the creep phenomenon occurring in asphalt binders modified with WS, taking into account the time-temperature equivalence principle.

In conclusion, the results of this study show an extremely significant effect of viscosity-reducing agents on the binder studied (especially at high pavement operating temperatures). The addition of synthetic wax to the reference bitumen increases the value of the dynamic modulus |G*| compared to the values obtained for the reference bitumen, thus contributing to the stiffening of the binder. The aging process plays an equally important role as the type of viscosity modifier. In the case of PMB 45/80-65 bitumen with synthetic wax, the long-term aging process reduces the load-time sensitivity of such a bitumen binder, in contrast to an asphalt binder with a surfactant which, after aging, becomes a load-time sensitive material (with a narrow relaxation spectrum) which quickly liquefies to become a viscous material.

### 3.5. Aging Characteristics of Asphalt Binders with SW Additives

In order to assess the influence of aging on the properties of PMB 45/80-65 with SW additive, the test results were standardized. Radar plots were made from the data obtained. The results are presented on a circular basis, with the quantitative values marked on lines radiating from a central point. This allows us to compare several variables simultaneously, all of which are equally relevant.

The characteristics that were standardized and shown on the radar charts were

- Penetration—Pen;
- Softening temperature—T_R&B_;
- Critical temperature obtained from the BBR test—T_CRIT_;
- Creep tendency J_nr_ at a stress level of 3.2 kPa—J_nr3.2_;
- Elastic rebound at a stress level of 3.2 kPa—R_3.2_.

All features were subjected to standardization through the well-recognized Z-score standardization method. This approach converts data values to achieve a mean of zero and a standard deviation of one, consistent with the following Formula (6):

(6) $$Z=\frac{\left(X-\mu \right)}{s}$$

where X is the experimental data point, μ is the mean of the dataset, and s is the standard deviation of the dataset. 

**Figure 15:**
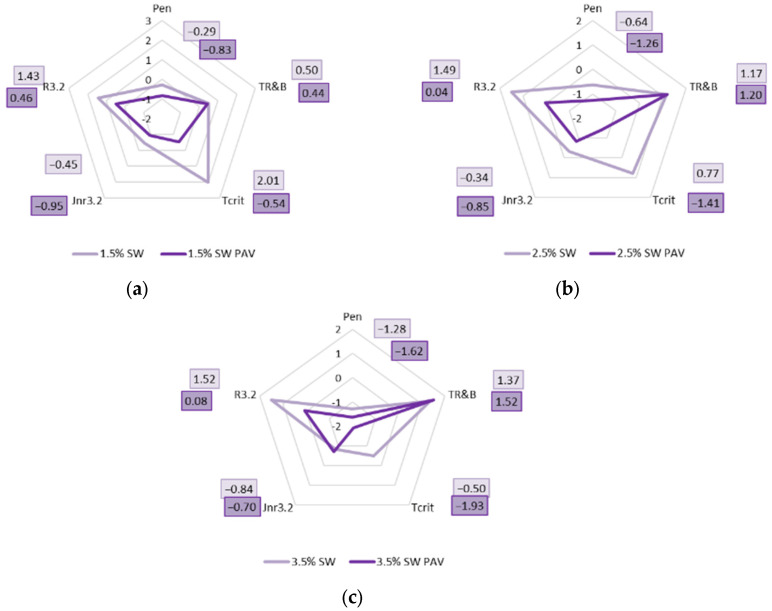
Influence of aging on selected standardized (Z-score) viscoelastic properties of PMB 45/80-65 bitumen modified with: (**a**) 1.5% SW, (**b**) 2.5% SW; (**c**) 3.5% SW.

Figure 15 shows radar plots showing the effect of long-term aging of PAV on PMB 45/80-65 polymer-modified bitumen with 1.5%, 2.5%, and 3.5% synthetic wax SW.

Upon analyzing the standardized results presented in the radar plots, it is evident that the long-term aging of PAV exerts the most pronounced impact on the critical temperature of the binder, as indicated by the substantial length of the vector of standardized results and the significant reduction in this parameter. A notable effect was also observed concerning the alteration in the values of creep compliance J_nr3.2_ and elastic recovery R_3.2_. The decrease in elastic recovery R_3.2_ correlates with the reduction in critical temperature, suggesting that the long-term aging process renders the binder modified with synthetic wax F-T more rigid and susceptible to brittleness. Moreover, it was identified that as the proportion of synthetic wax in the binder increases, the discrepancies in the values of J_nr3.2_ progressively diminish, culminating in a scenario where, at a wax content of 3.5%, the creep compliance after PAV aging surpasses that observed before aging. Furthermore, the process of long-term aging appears to have a negligible impact on the softening point values of the binders. Additionally, the variations in the obtained penetration values remain minimal.

At this juncture, it is plausible to propose the adoption of the MSCR method within the existing EN standard for modified bitumens, aimed at assessing the aging process of asphalt binders. This method provides significantly greater variability and a more comprehensive understanding of the binder’s behavior, compared to, for instance, the softening temperature of the binder.

### 3.6. Influence of the SW Additive on the Aging of PMB 45/80-65

**Figure 16:**
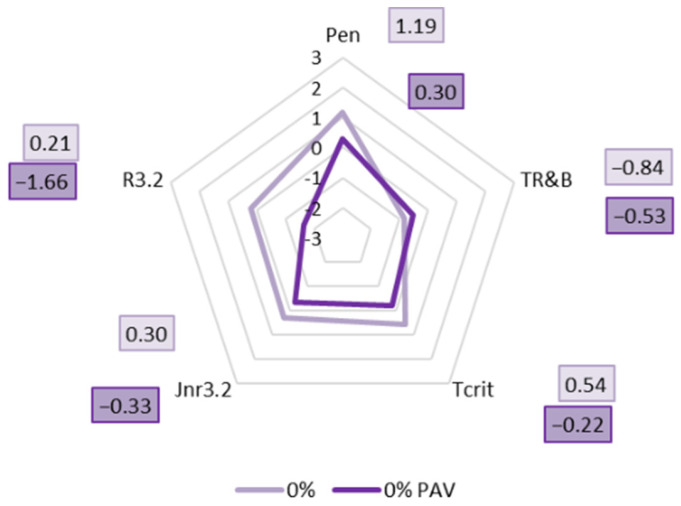
Changes in the standardized (Z-score) properties of PMB 45/80-65 before and after PAV aging.

In order to provide a comprehensive picture of the effect of the synthetic wax on the aging of PMB 45/80-65, radar plots showing the changes in binder properties were made using all the standardized data obtained previously. Figure 16 shows a radar plot showing the effect of long-term aging of PAV on PMB 45/80-65 without viscosity-reducing additives.

Based on the analysis of the standardized test results shown in Figure 16, it can be concluded that, in the case of PMB 45/80-65 without SW addition, the PAV aging process has the greatest effect on the elastic recovery value at a stress of 3.2 kPa (the difference in the standard deviation value is the greatest). A significant decrease in the value of R_3.2_ is noticeable, indicating a significant stiffening of the binder as a result of the PAV aging process, which is in agreement with the observations [68,69,70].

Analyzing the data presented in Figure 16, it can also be seen that the aging process causes a slight decrease in penetration values with a slight increase in the softening temperature of PMB 45/80-65 bitumen. A decrease in the critical temperature T_crit_ and in the J_nr3.2_ was also observed. This indicates that the binder becomes more brittle and friable as a result of the RTFOT+PAV long-term aging process.

**Figure 17:**
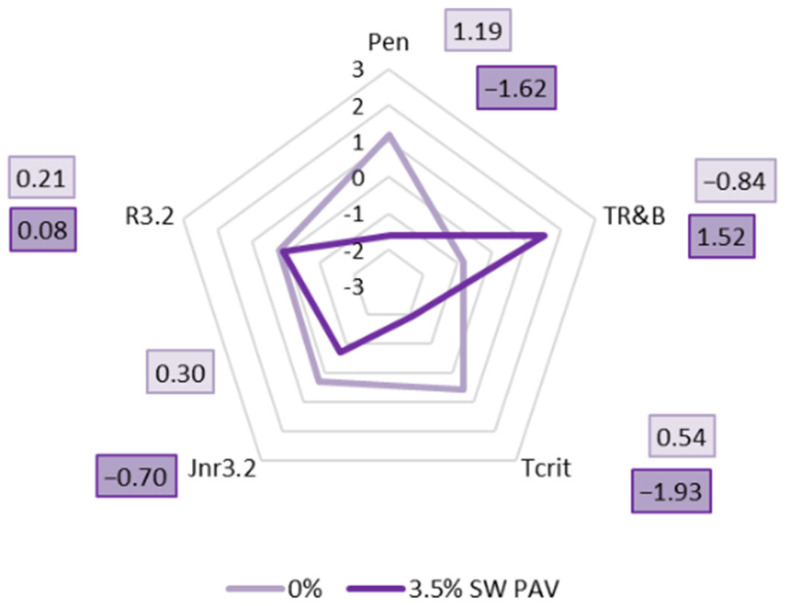
Change in standardized (Z-score) properties of PMB 45/80-65 bitumen before aging and with 3.5% SW synthetic wax added after PAV aging.

On the other hand, Figure 17 shows a radar plot showing the changes in the properties of PMB 45/80-65 bitumen before aging and PMB 45/80-65 bitumen with the addition of 3.5% synthetic wax after aging PAV.

From the analysis of the data presented in Figure 16, it can be concluded that the long-term aging of PAV has had the greatest effect on the binder penetration value (the length of the result vector on the scale subjected to standardization is the greatest, and there is the greatest decrease in this value). At the same time, a significant increase in the T_R&B_ value of the 3.5% SW PMB 45/80-65 bitumen can be seen in the above radar plot. After aging, it is likely that there has been an increase in molecular weight and condensation of aromatic hydrocarbons in the binder, which is reflected in the penetration value and softening point.

The addition of synthetic wax to PMB 45/80-65 also had a significant effect on the critical temperature of the binder. A significant increase in the value of this parameter was observed after long-term aging of PAV. This indicates that the additive affects the stiffening of the polymer-modified bitumen, which contributes to the creep compliance of the pavement to low-temperature cracking.

The parameters obtained from the MSCR test—creep compliance and elastic return at a stress of 3.2 kPa—changed to a lesser extent. The long-term aging process had a negligible effect on the R_3.2_ value. However, a decrease in J_nr3.2_ was observed, confirming earlier conclusions that polymer-modified bitumen becomes a more elastic material with the addition of synthetic wax. It can also be assumed that the resistance to permanent deformation of a mineral asphalt mix with such a binder will be higher.

### 3.7. Visualization of the Spectrum of Asphalt Binders in an Epi-Fluorescence Microscope

To complement the analysis of the study of the rheological properties of PMB 45/80-65 bitumen with the addition of SW, their spectrum is presented in photographs taken with an epi-fluorescence microscope according to EN 13632. The observations were made at 100 times magnification of the bitumen binder sample at 25 °C. When the polymer-modified bitumen is illuminated with UV ultraviolet light using optical filters, a difference in the luminescence of the polymer and bitumen phases is noticeable. The bitumen matrix provides a dark brown to black image. The polymer dispersed in the bitumen matrix can be observed as a light yellow or yellow dispersed phase with varying degrees of dispersion.

**Figure 18:**
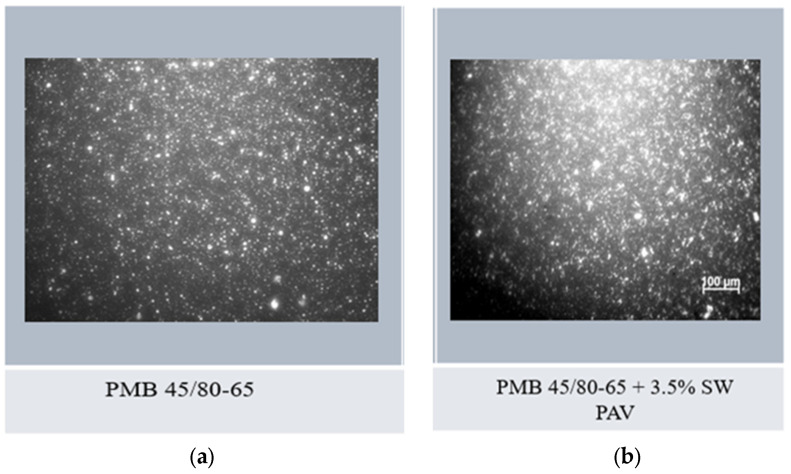
Epi-fluorescence spectra of bitumen binders (M. Cholewińska): (**a**) SBS modified bitumen PMB 45/80-60; (**b**) SBS modified bitumen PMB 45/80-60 + 3.5% SW after PAV.

In order to best demonstrate the changes that occur after long-term aging of PAV in bitumen binders with viscosity reducing agents, Figure 18 shows three photographs taken with an epi-fluorescence microscope:

- Reference bitumen PMB 45/80-65 unaged (Figure 18a);
- Reference bitumen PMB 45/80-65 with synthetic wax SW added at 3.5% after PAV (Figure 18b).

Observation of the microstructure in the form of a spectrum of the reference bitumen PMB 45/80-65 (Figure 18a) under a light microscope revealed uniformly dispersed small polymer particles in a continuous bitumen phase. On the other hand, the analysis of the spectrum shown in Figure 18b, corresponding to the bitumen PMB 45/80-65 with the addition of 3.5% SW after the PAV aging process, revealed crystallites of synthetic wax between the polymer particles. The presence of the crystalline phase of the wax used suggests that the addition of the modifier acts as a fine crystalline filler [11]. This results in an increase in the stiffness of the asphalt binder through the formation of an internal crystalline structure. The observation of the spectrum of the PMB 45/80-65 bitumen modified with the synthetic wax SW after the PAV aging process in the fluorescence microscope and the conclusions drawn from it are in agreement with the results of the study of the rheological properties of the asphalt binder presented previously.

## 4. Conclusions

On the basis of the FRTOT and PAV tests carried out on PMB 45/80-65 with the addition of SW synthetic wax, the following conclusions can be formulated:

1. The incorporation of synthetic wax (SW) into PMB 45/80-65 binder facilitates the production of bitumen with significantly enhanced properties at both high and low temperatures. The optimal dosage range of 2% ± 0.5% (m/m) ensures a total increase in the plasticity index by 9 °C to 11 °C, more than a twofold decrease in susceptibility to rutting (J_nr3.2_ ≤ 0.35 kPa^−1^), and maintenance of low thermal sensitivity (PI ≈ +3).
2. The results of the zero shear viscosity η_0_ and the parameter |G*|/sinδ indicate an increase in the thixotropic character of the bitumen modified with 3.5% synthetic wax. This effect is related to the appearance of a large number of synthetic wax crystallites in combination with the presence of the polymer in the bitumen, which probably disturbs the state of colloidal equilibrium.
3. The SW addition ≤ 2.5% does not significantly deteriorate T_crit_, which is advantageous for resistance to low-temperature cracking.
4. Observations conducted utilizing an epi-fluorescence microscope revealed that crystalline SW functions as a fine-crystalline filler, thereby augmenting the strength of the binder.
5. The aging process of bitumen results in reductions in PI and R_3.2_. However, this reduction is less pronounced when a higher SW value is incorporated into the bitumen. This observation confirms that synthetic wax serves to partially counteract the degradation of polymers.
6. The Christensen Anderson Marasteanu (CAM) model was fitted to the results of an experiment designed to simulate the aging process of bitumen containing a viscosity reducing SW additive. It was shown to be a very good fit to the experimental data, suggesting that it is a good mathematical tool for describing the creep phenomenon occurring in bitumen, taking into account the equivalence of time and temperature.

In conclusion, this composite material is suitable for practical applications, enabling lower asphalt mixing and paving temperatures, which reduces energy consumption and emissions. It improves aging resistance and rheological properties in polymer-modified bitumen when combined with Sasobit. Its durability and reduced viscosity are ideal for urban areas needing minimized pollution and energy use. The material has potential to enhance the sustainability, durability, and performance of asphalt pavements, particularly in applications demanding lower energy use and extended service life.

## Figures and Tables

**Table 1:** Basic physical and mechanical properties of PMB 45/80-65 bitumen.

Property	Test Method	Unit of Measure	Result
Penetration at 25 °C	EN 1426	0.1 mm	45.3
Softening point T_R&B_	EN 1427	°C	74.7
Fraass breaking point	EN 12593	°C	−17.8
Cohesion Energy at 5 °C	EN 12591	J/cm^2^	6.4
Elastic recovery at 25 °C	EN 12591	%	87
Dynamic viscosity w 60 °C:	EN 12591	Pas	645

**Table 2:** Characteristics of the synthetic wax SW [35].

Property	Unit of Measure	Value
Appearance	-	solid pellets, white, or yellowish
Flash point	°C	285
Solidification point	°C	95
Density at 25 °C	Mg/m^3^	0.9
Molecular weight	g/mol	ca. 1000

**Table 3:** Experiment matrix.

SW [% m/m]	Virgin	RTFOT	PAV (RTFOT + PAV)
0.0	C R P M	C R P	C R P
1.5	C R P M	C R P	C R P
2.5	C R P M	C R P	C R P
3.5	C R P M	C R P	C R P M

Test legend: C—conventional testing (penetration, T_R&B_, T_Fraass_, PI, PR). R—rheological tests (DSR test at temperature sweep 40 °C ÷ 80 °C, zero shear viscosity η_0_). P—performance tests (MSCR test at 60 °C, BBR test such as S, m, Tcrit). M—epi-fluorescence microscopy.

**Table 4:** Results of fitting the CAM model at 60 °C.

Modifier Type	Additive Amount (%)	Aging Treatment	CAM Model Parameters			Rheological Index *R*	R^2^
			*f_c_* [Hz]	*v*	*w*		
Reference	0	Unaged	6838.65	0.366039	0.777134	0.822398	0.99
SW	1.5	Unaged	4463.62	0.128138	0.681133	2.349270	0.99
SW	2.5	Unaged	56,041.80	0.236811	0.666271	1.271181	0.99
SW	3.5	Unaged	64,110.16	0.181204	0.492674	1.661274	0.99
Reference	0	RTFOT	3279.24	0.118217	0.669327	2.546422	0.99
SW	1.5	RTFOT	3389.39	0.152007	0.634313	1.980375	0.99
SW	2.5	RTFOT	51,721.91	0.307923	0.504736	0.977616	0.99
SW	3.5	RTFOT	82,890.49	0.070345	0.344391	4.279335	0.99
Reference	0	RTFOT+PAV	3664.013	0.164292	0.784387	1.832292	0.99
SW	1.5	RFTOT+PAV	22,612.67	0.270815	0.586265	1.111572	0.99
SW	2.5	RFTOT+PAV	88,153.27	0.099749	0.379157	3.017875	0.99
SW	3.5	RFTOT+PAV	33,343.37	0.105717	0.447592	2.847504	0.99

## Data Availability

The original contributions presented in this study are included in the article. Further inquiries can be directed to the corresponding author(s).
